# Round-shape gold nanoparticles: effect of particle size and concentration on *Arabidopsis thaliana* root growth

**DOI:** 10.1186/s11671-018-2510-9

**Published:** 2018-04-10

**Authors:** Jakub Siegel, Kamil Záruba, Václav Švorčík, Kristýna Kroumanová, Lenka Burketová, Jan Martinec

**Affiliations:** 1 Department of Solid State Engineering, University of Chemistry and Technology, 166 28 Prague, Czech Republic; 2 Department of Analytical Chemistry, University of Chemistry and Technology, 166 28 Prague, Czech Republic; 3 Institute of Experimental Botany of the Czech Academy of Sciences, 165 02 Prague, Czech Republic

**Keywords:** Gold nanoparticles, Size, Concentration, *Arabidopsis thaliana*, Root growth

## Abstract

Nowadays, due to a wide range of applications of nanoparticles (NPs) in many industrial areas, accumulations of those entities in environment pose a great risk. Owing to their inertness, noble metal NPs may remain in contaminated soils nearly unchanged for long time. Within this context, size-, shape-, and concentration-dependent uptake of particles by plants belongs to unexplored area. In this work, we present water solutions of biologically friendly synthesized spherical AuNPs with pretty narrow size distribution in size range from 10 to 18 nm. Their thorough characterization by atomic absorption spectroscopy, mass spectroscopy-equipped inductively coupled plasma, dynamic light scattering (DLS), and TEM methods was followed by the study of their effect on the growth of *Arabidopsis thaliana* (primary and lateral roots), in particle size- and concentration-dependent manner. Due to strictly round-shape form of AuNPs and absence of particle agglomeration, DLS-derived size and size distribution were in good concordance with those obtained from TEM. The length and number of *A. thaliana* lateral roots were significantly affected by all types of AuNPs. Smallest AuNPs at highest concentration inhibited length of primary roots and, in contrast, enhanced hair root growth.

## Background

Nowadays, modern chemistry and engineering churn out huge amounts of nanoobjects in order to improve utility properties of matter not only in special applications but increasingly in products of everyday consumption. Nanostructured materials, i.e., nanoparticles [1, 2], nanorods [3], nanotubes [4], nonwoven nanotextiles [5], as they stand or attached to different kinds of supports, significantly increase yields in almost all areas of industrial applications ranging from cosmetics [6] and health care [7], over bioengineering [8, 9], up to energy conversion applications [10], and catalysts [3]. While the inclusion of nanomaterials in these products can enhance their performance, their breakdown at the end of their useful lifetime provides several key points of entry for synthetic NPs into the environment. Especially engineered NPs, which has been extensively used in catalysts, UV-protective dye stabilizers, antimicrobial agents in textile industry, or health care products and cosmetics (in particular that of high chemical inertness such as Au, Ag, Pt, and Pd) must be given special attention, since they may accumulate in the environment almost unchanged for many years, triggering so far unknown processes upon their plant uptake. Concerning noble metal nanoparticles (NMNPs), some pioneering works has been published on the effect of silver nanoparticles (AgNPs) on seedlings of thale cress (*Arabidopsis thaliana*), indicating that very low concentrations of AgNPs (< 1 ppm) could be toxic to the seedlings [11]. AgNPs of 20 to 80 nm clearly stunted the growth, and their phytotoxicity is concentration and particle size dependent. The root tip (cap and columella) were observed to turn light brown when primary roots were exposed to AgNPs. The brown tip was attributed to the adsorption of AgNPs either itself or in conjunction with cell wall materials or secondary metabolites produced by root tips. However, the exact mechanism has not been elucidated yet.

Even though there have been some studies addressing the role of NPs in the environment [12], those targeting on the gold nanoparticles (AuNPs) are still rare [13]. If available, the majority of the published data on nanotoxicology have focused on mammalian cytotoxicity [14–16] or impacts to animals and bacteria [17–20], and only a few studies have considered the toxicity of engineered NPs to plants. Additionally, the interaction of NMNPs with plants and other organisms that share similarities with plant cells, such as algae, have been poorly studied so far, implying that the general consequences of NMNPs exposure for plant cells still remains unclear [11]. The lack of these data leads to a defective understanding of how NMNPs are transferred and accumulated in the various food chain levels.

In this work, we report on the effect of gold nanoparticles on plant growth, particularly on the development of primary and lateral roots of *A. thaliana* in presence of different size particles. AuNPs were synthesized by wet method under biologically friendly protocol using no stabilizers, producing spherical nanoparticles with precise control over their size and size distribution. Before the plant treatment, AuNPs were thoroughly characterized by a broad spectrum of analytical methods (AAS, ICP-MS, DLS, and TEM).

## Experimental

### Materials, apparatus, and procedures

Gold nanoparticles were synthesized by a slightly adapted procedure published by Batús et al. [20]. Briefly, 149 mL of water was heated in a 250 mL two-necked round-bottomed flask until it starts to reflux. Then, 1 mL of 0.33 M sodium citrate and 0.945 mL of 10 mg/mL of potassium tetrachloroaurate(III) in water were subsequently added. After 30 min, heating was stopped and the reaction mixture was left to get cold. In all preparation experiments, Milli-Q water (18.2 MΩ at 25 °C) was used.

For root assays of *A. thaliana*, synthesized AuNPs of three different sizes (10, 14, and 18 nm) were centrifuged at 5000*g* for 1 h to increase particle concentration up to limit value of 2000 mg/L.

*A. thaliana* Columbia (Col-0) seeds (obtained from Lehle seeds, USA) were surface sterilized with 30% (*v*/*v*) bleach solution for 10 min and rinsed five times with sterile water. Sterile seeds were sown on agar plates containing ½ Murashige-Skoog (MS) medium and 1% plant agar (pH 5.8). To synchronize seed germination, the agar plates were kept at 4 °C for 2 days. *A. thaliana* plants were grown for 5 days in vertically oriented plates in a growth chamber at 22 °C with 100 μmol m^− 2^ s^− 1^ light intensity under long day conditions (16 h/8 h light/dark cycle).

Five-day-old seedlings of similar size were transferred to agar plates (20 plants per plate) containing 1/16 MS medium, different concentrations of AuNPs (0, 1, 10, and 100 mg/L) and 1% plant agar (pH 5.8). AuNPs were added to the medium after autoclaving. As a control, the effect of sodium citrate buffer was also investigated. The length of the root was marked, and seedlings were grown for the next 5 days. Both, increment of the primary root and lateral roots length were measured using JMicroVision 1.2.7 software.

### Analytical methods

Prepared solutions of AuNPs were characterized by Atomic Absorption Spectroscopy (AAS), mass spectroscopy-equipped inductively coupled plasma (ICP-MS), dynamic light scattering (DLS), and transmission electron microscopy (TEM).

Concentrations of prepared NPs were determined by means of AAS by a VarianAA880 device (Varian Inc., USA) using a flame atomizer at 242.8 nm wavelength. Typical uncertainty of concentration determined by this method is less than 3%.

Inductively coupled plasma with mass spectroscopy detector (ICP-MS) was used to determine the concentration of Au ions originating from unreacted Au source chemical, using Agilent 8800 triple-quadrupole spectrometer (Agilent Technologies, Japan) connected to an auto-sampler. AuNPs colloid solution was pipetted into 1.5 mL hydrophobic microtubes and centrifuged at 30000*g* on Eppendorf 5430 centrifuge for 1 h. After the centrifugation, 0.3 mL of supernatant was carefully removed using pipette and ICP-MS analyzed. Sample nebulization was performed using a MicroMist device equipped with a peristaltic pump. Pure buffer solution (2.2 mM sodium citrate) was used as a blank sample. The uncertainty of the measurement was less than 3%.

TEM images were measured using JEOL JEM-1010 (JEOL Ltd., Japan) operated at 400 kV. Drop of colloidal solution was placed on a copper grid coated with a thin amorphous carbon film on a filter paper. The excess of solvent was removed. Samples were air dried and kept under vacuum in a desiccator before placing them on a specimen holder. Particle size was measured from the TEM micrographs and calculated by taking into account at least 500 particles.

The particle size distribution was determined by Zetasizer ZS90 (Malvern Instruments Ltd., England) in the DLS regime for particle size distribution, equipped with an avalanche photodiode for signal detection. Diode-pumped solid state laser (50 mW, 532 nm) was used as a light source. The measurements were performed in polystyrene cuvettes at room temperature.

## Results and discussion

### Nanaoparticle characterization

Size and size distribution of AuNPs were determined by TEM and DLS analyses. The results are summarized in Table 1, together with AuNP concentrations determined by AAS immediately after NPs synthesis and concentrations of residual Au ions determined by ICP-MS. From these data, it is evident that our synthesis protocol provides well size-controlled Au nanoparticles with pretty narrow size distribution. Here, we used modified method published by Batús et al. [20] for the synthesis of size- and shape-controlled citrate-stabilized AuNPs. Developed protocol allows targeted size-extension based on enlargement of pre-synthesized AuNPs by means of surface-catalyzed reduction of Au^3+^ together with effective simultaneous inhibition of secondary nucleation.

**Table 1:** The size and size distribution of AuNPs obtained by TEM and DLS analysis together with concentrations of AuNPs (AAS) and Au ions (ICP-MS)

	NP size (nm)		Concentration of AuNPs/Au ions (μg/L)		
	TEM	DLS	AuNPs (× 10^−3^)	Au ions^a^	Au ions^b^
10 nm	11.8 ± 2.7	9.6 ± 3.7	30.2 ± 0.9	339.8 ± 27.4	1.6 ± 0.3
14 nm	15.1 ± 3.8	14.3 ± 3.9	29.8 ± 0.8	328.6 ± 24.1	2.1 ± 0.5
18 nm	19.6 ± 3.2	18.1 ± 4.1	29.8 ± 0.8	314.9 ± 29.9	1.8 ± 0.4

^a^Immediately after the synthesis

^b^After centrifugation of as-synthesized AuNPs and its dilution to probing concentration of 100 mg/L

Wang et al. [21] found that during a 3-day hydroponic exposure of *A. thaliana* to either Ag^+^ and AgNPs (5 nm) at the same concentration, the Ag concentration decreased faster in the Ag^+^-treated solution than in the AgNP one, indicating faster uptake of Ag^+^ ions. Therefore, we took special attention to minimize the possible effect of Au ions in result distortion. As-synthesized AuNPs were centrifuged up to limit concentration of 2000 mg/L and diluted with MS medium to required concentrations (1, 10, and 100 mg/L). After this procedure, the concentration of residual Au ions in solutions containing 100 mg/L of AuNPs was determined by ICP-MS (see Table 1). Apparently, the centrifugation had positive effect on both the presence of residual Au ions which concentrations were reduced by two orders of magnitude compared to as-synthesized solutions (Table 1, AAS) and the content of citrate buffer itself.

**Fig. 1:**
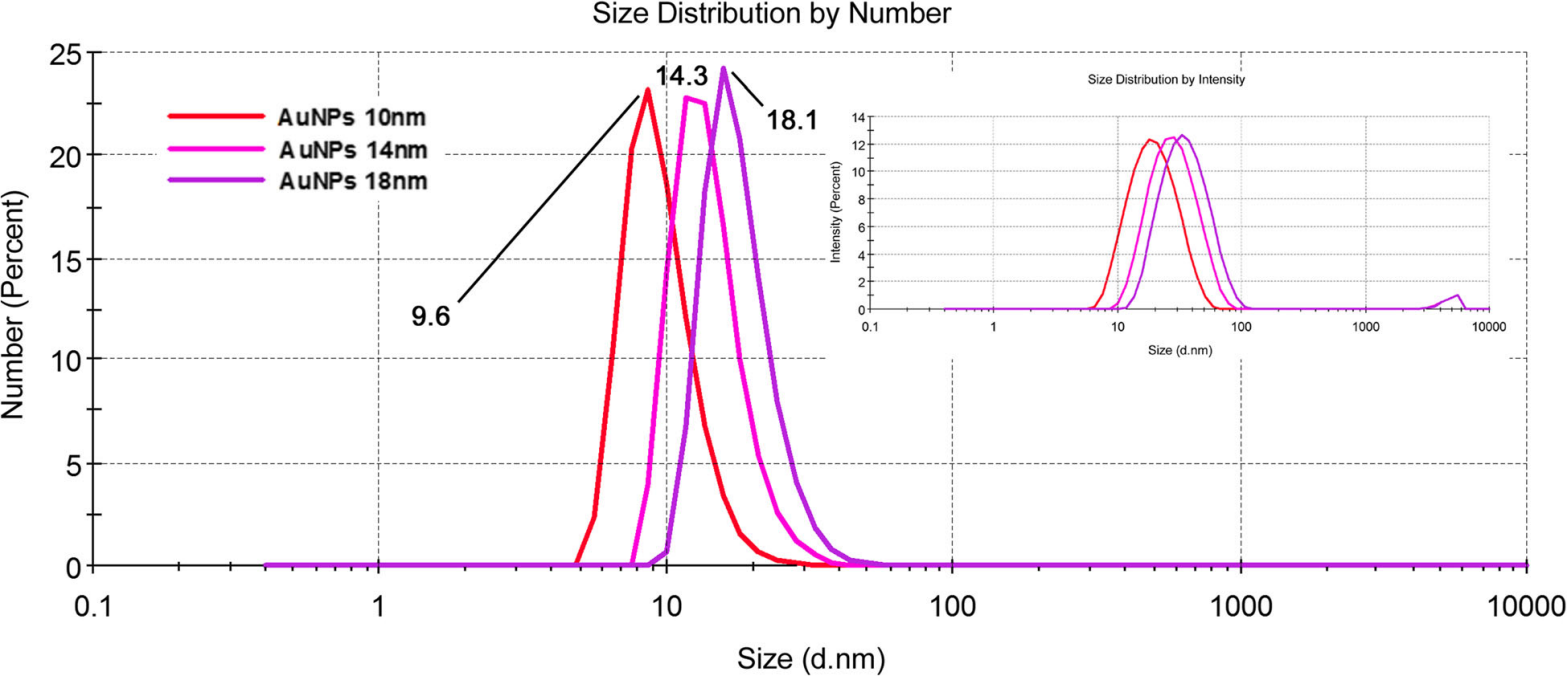
Dynamic light scattering analysis (number weighted size distribution) of aqueous solutions of AuNPs of different sizes. Inset shows “raw” intensity-weighted data. Numbers refer to average particle diameter in nm

**Fig. 2:**
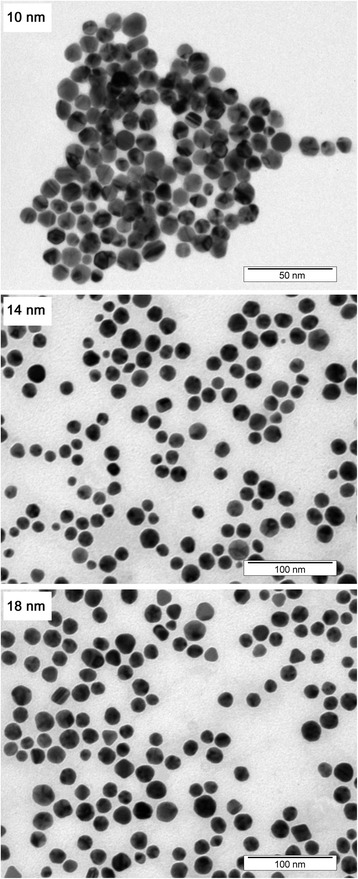
TEM images of prepared set of AuNPs. Please note that the picture magnification differs for particular NPs sizes

To quantify NP polydispersity, we performed DLS measurement, which is very sensitive to the presence of eventually formed particles conglomerates (Fig. 1). In this measurement, even a small amount of agglomerated NPs causes the dominance of corresponding peak at considerably higher diameters, especially in the intensity-weighted size distribution (see inset in Fig. 1). Fortunately, no particle agglomeration was detected, and thus, the credibility of the size evaluation may be affected only by spherical approximation [22]. Despite this disadvantage, DLS measurement provides statistically more significant overall picture of particle size distribution compared to TEM, since it evaluates the entire sample volume at once. Due to predominately round-shaped nature of prepared NPs (see Fig. 2), DLS-derived sizes were in good agreement with those obtained by TEM (Table 1). Apparent particle agglomerates, visible in TEM image (Fig. 2, 10 nm), were more likely caused by the necessity of solvent removal in TEM measurements, rather than interconnection of individual particles in colloid solution itself.

### Adaptation of nanoparticle solutions to meet plant biology conditions

Due to strong aggregation tendency of AuNPs in plant growth media (MS medium), commonly used for plant growth in vitro [23], we had to optimize mutual ratio of these two components to avoid NPs aggregation while preserving acceptable conditions for plant growth. Different dilutions of MS were tested. Aggregation of AuNPs was easily visible by the change of the color (transition from red to purple). Due to low initial concentration of as-synthesized AuNPs (around 30 mg/L, see Table 1), insufficient for biological experiments, it was necessary to increase the NP concentration by centrifugation. By this procedure, we increased particle concentration up to the limit value of 2000 mg/L. Such NPs solutions were then diluted to final concentrations, which also decreased the concentration of the citrate buffer in our experiments. For growing experiments of *A. thaliana*, we diluted AuNPs in 1/16 MS medium. Minimal NPs aggregation was detectable in this medium and NPs were much more stable in comparison to 1/2 MS, 1/4 MS, and 1/8 MS with nearly unaltered plant growth. Concentration of AuNPs in final solutions was determined by AAS. Since plant growth experiments were performed in in vitro (in sterile conditions), the effect of sterilization procedure on NPs was studied too. Commonly used autoclaving (121 °C, 20 min) of prepared growth media caused complete aggregation of investigated NPs. Therefore, this procedure was not suitable for our experiments. Addition of NPs into about 60 °C autoclaved agar medium was finally used as an alternative procedure in which no NP aggregation was detected and sterilizing process was still effective.

### Effect of NPs on the root growth of *Arabidopsis thaliana* in vitro

**Fig. 3:**
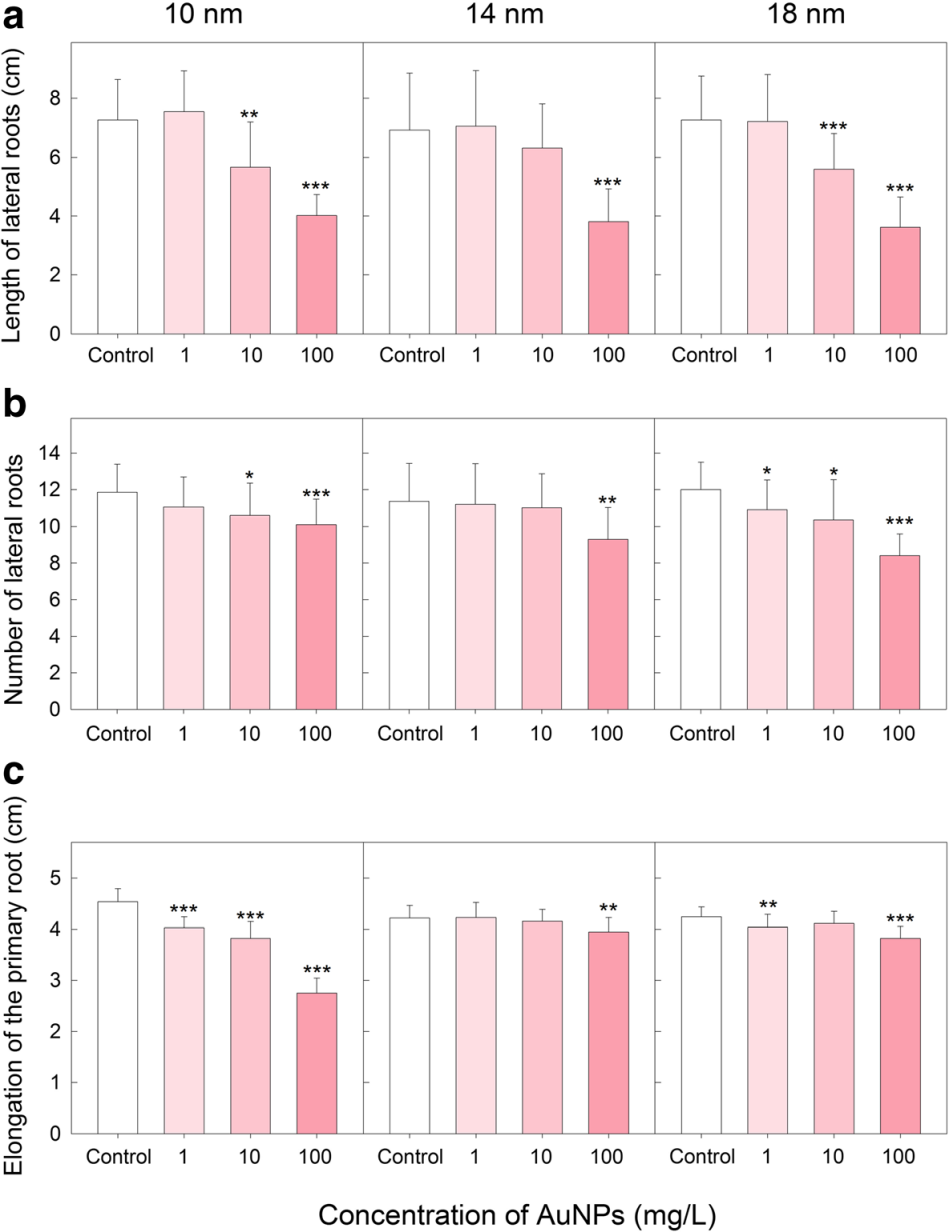
Effect of AuNPs on **a** length and **b** number of lateral roots and **c** elongation of the primary root of *A. thaliana* seedlings. Plants were exposed to different concentrations (0.1, 10, and 100 mg/L) of 10, 14, and 18 nm AuNPs. Data are means + SD of 19–20 plants. *P < 0.05, **P < 0.01, ***P < 0.001; *t* test

Model plants such as dicot *A. thaliana* have the potential to help understand stress factors that reduce global crop yields with the goal of identifying genes that can improve the viability under stress conditions [24]. All tested forms of AuNPs had significant effect on lateral roots (LRs). Both the length (Fig. 3a) and the number (Fig. 3b) of LRs were reduced in AuNP-treated plants. The highest concentrations of AuNPs (100 mg/L) of all investigated particle sizes induced decrease of LRs length to about 50%. Number of LRs decrease to about 70% in the case of 18 nm AuNPs and the highest concentration (100 mg/L). Slightly smaller reduction of number of LRs was observed when the highest concentration of smaller AuNPs (14 and 10 nm) was used (Fig. 3b). The length of primary roots was decreased after AuNP treatment as well (Fig. 3c). The negative effect of 10-nm AuNP was considerable especially at higher particle concentrations. Effect of bigger particles (14, 18 nm) was much smaller and similar to the effect of sodium citrate buffer that was used as control. Most published studies with engineered NPs indicated certain degree of phytotoxicity, especially at high NP concentrations. For instance, citrate-coated AgNPs inhibited *A. thaliana* seedling root elongation with a linear dose response from 67 to 535 μg/L after 2 weeks [25]. Several other studies using the root elongation and seed germination assays have demonstrated that phytotoxicity was affected by the size of NPs. Many studies have concluded that the smaller the NPs the more phytotoxic they are. However, this generalization about the size-dependent toxicity of engineered nanomaterials is not always true for all combinations of plants and NPs types [21, 25]. Contrary to that, single-wall carbon nanotubes positively affected root elongation of tomato, cabbage, carrot, and lettuce in 24 to 48 h [26]. Positive effect of 24 nm AuNPs on seed germination rate and vegetative growth was reported by Kumar et al. [13].

**Fig. 4:**
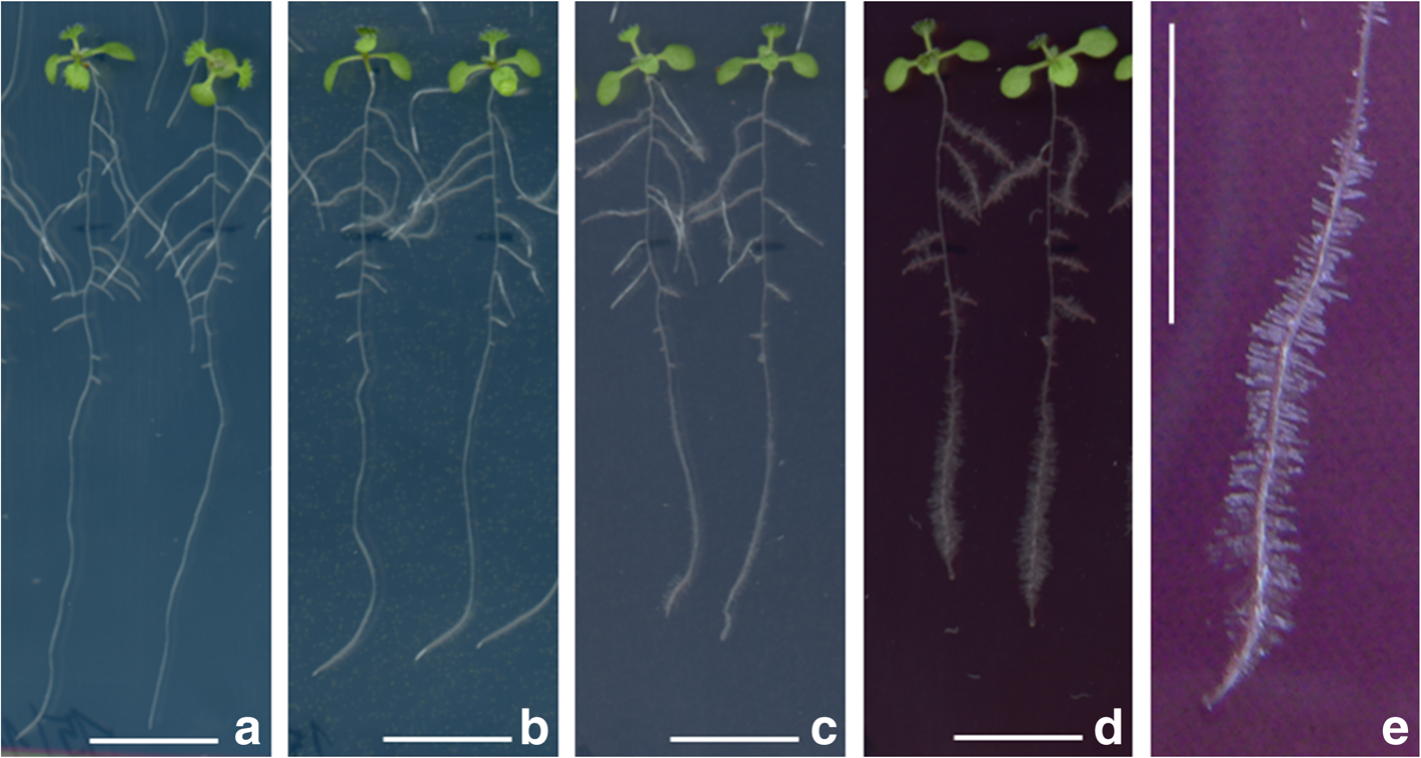
Effect of different concentrations of 10 nm AuNPs on root hair growth in *A. thaliana* seedlings. **a** Control, **b**–**d** seedlings treated with 1, 10, and 100 mg/L of AuNPs, respectively, and **e** detail of induced root hair growth in plant exposed to 100 mg/L of AuNPs. The scale bars correspond to 1 cm

Substantial positive effect of 10 nm AuNPs on a root hair growth was observed during root growth experiments (Fig. 4). This effect exhibited strong concentration dependency. Increasing NP concentration induced more pronounced growth of root hairs (Fig. 4e). This behavior has been often observed in root grown in soils poor in phosphorous [27]. A similar effect was not observed in the case of 14 and 18 nm AuNPs. Contrary to that, García-Sánchez et al. [28] observed, during *A. thaliana* treatment with commercially available AgNPs, inhibition in a number of root hairs related to 1 cm of plant root. Decrease in hair roots was observed in the case of all tested particles, regardless of their specific size (10, 20, 40, and 80 nm), using a uniform concentration of treatment solutions of 200 mg/L. Root hairs substantially increase the root surface area in contact with the soil, and most of water and nutrients that enter the plant are absorbed through them. Thus, their development is significantly affected by environment stimuli and stress signals [29].

## Conclusions

We have successfully prepared gold nanoparticles by gentle two-component (sodium citrate–potassium tetrachloroaurate) reduction in water environment providing round-shaped, narrow-distributed particles with excellent control over their resulting size. Post-synthesis centrifugation allowed to reach the desired NPs concentrations and eliminated the influence of ions and citrate buffer on results distortion in plant experiments. The effect of AuNPs of different sizes (10, 14, and 18 nm in diameter) and concentrations (1, 10, and 100 mg/L) on root growth of *A. thaliana* was investigated. The number and length of lateral roots were significantly decreased after the treatment with NPs solutions of higher particles concentrations, regardless of their specific size. A negative effect on primary root growth was observed in the case of 10 nm AuNPs. Surprisingly, the smallest AuNPs (10 nm) clearly induced root hair growth. Overall, this study showed that the direct exposure of plants to AuNPs significantly contributed to phytotoxicity and underscores the need for eco-responsible disposal of wastes and sludge containing Au nanoparticles.

## Abbreviations

AAS: Atomic absorption spectroscopy
AgNPs: Silver nanoparticles
AuNPs: Gold nanoparticles
DLS: Dynamic light scattering
ICP-MS: Mass spectroscopy equipped inductively coupled plasma
LRs: Lateral roots
MS: Murashige and Skoog
NMNPs: Noble metal nanoparticles
NPs: Nanoparticles
TEM: Transmission electron microscopy
